# Quantitative susceptibility mapping reveals differences between subtypes of Lewy body dementia

**DOI:** 10.1093/brain/awaf325

**Published:** 2025-09-04

**Authors:** Rohan Bhome, George E C Thomas, Naomi Hannaway, Ivelina Dobreva, Angeliki Zarkali, Karin Shmueli, James H Cole, Rimona S Weil

**Affiliations:** Dementia Research Centre, University College London, London WC1N 3AR, UK; UCL Hawkes Institute, University College London, London WC1V 6LJ, UK; Dementia Research Centre, University College London, London WC1N 3AR, UK; Dementia Research Centre, University College London, London WC1N 3AR, UK; Dementia Research Centre, University College London, London WC1N 3AR, UK; Dementia Research Centre, University College London, London WC1N 3AR, UK; Department of Medical Physics and Biomedical Engineering, University College London, London WC1E 6BT, UK; Dementia Research Centre, University College London, London WC1N 3AR, UK; UCL Hawkes Institute, University College London, London WC1V 6LJ, UK; Dementia Research Centre, University College London, London WC1N 3AR, UK; Wellcome Centre for Human Neuroimaging, University College London, London WC1N 3AR, UK; Movement Disorders Centre, National Hospital for Neurology and Neurosurgery, London WC1N 3AR, UK

**Keywords:** Lewy body dementia, quantitative susceptibility mapping, Parkinson’s disease dementia, dementia with Lewy bodies

## Abstract

Dementia with Lewy bodies (DLB) and Parkinson’s disease dementia (PDD) are subtypes of Lewy body dementia, and are considered two ends of a disease spectrum. However, conventional MRI neuroimaging, mainly focused on grey matter volume and thickness, has failed to establish whether underlying processes differ between them. Understanding these differences could enable targeted and subtype-specific treatments to be developed.

We applied quantitative susceptibility mapping (QSM), an advanced neuroimaging technique sensitive to tissue iron, to examine differences in tissue composition between these Lewy body dementia subtypes. We performed both voxel-wise and region of interest analyses to compare QSM values in 66 people with Lewy body dementia (45 DLB; 21 PDD), 86 people with Parkinson’s disease with normal cognition (PD-NC) and 37 healthy controls. We also assessed relationships between QSM values and measures of both cognitive performance and overall disease severity in people with Lewy body dementia.

We found that people with Lewy body dementia had higher QSM values in widespread brain regions, compared with cognitively normal people with PD, and that people with PDD had higher QSM values across many brain regions, compared with people with DLB. Further, we showed a positive relationship between QSM values and overall disease severity, measured using the Movement Disorder Society Unified Parkinson’s Disease Rating Scale in people with Lewy body dementia, in the right thalamus, left pallidum, bilateral substantia nigra, bilateral middle frontal, temporal and lateral occipital lobes, right precentral and superior frontal cortices. In a region of interest analysis, we showed that people with PDD had higher QSM values than cognitively normal people with PD and controls in the substantia nigra pars reticulata.

Our findings indicate neurobiological differences between subtypes of Lewy body dementia, that can be detected by exploiting QSM’s sensitivity to tissue composition. Based on this, DLB and PDD could be considered as distinct conditions in the clinic and in clinical trials, and may respond to different treatments. Our finding that QSM values relate to real-world measures of overall disease severity in Lewy body dementia indicates its potential as an imaging biomarker for clinical trials of Lewy body dementia interventions.

## Teaser

Using quantitative susceptibility mapping, an advanced neuroimaging technique sensitive to tissue iron, Bhome *et al.* identify neurobiological differences between Parkinson’s disease dementia and dementia with Lewy bodies. Their findings support treating these conditions as distinct in both clinical care and research.

## Introduction

Lewy body dementia is an umbrella term that comprises both dementia with Lewy bodies (DLB) and Parkinson’s disease dementia (PDD). Together, these conditions are the second commonest neurodegenerative dementia in people aged over 65 years, conferring significant morbidity and healthcare burden.^1^ They are characterized by dementia affecting visuospatial, executive and attentional function, and the presence of at least two symptoms of motor Parkinsonism, visual hallucinations, cognitive fluctuations and rapid eye movement (REM) sleep behaviour disorder. DLB is diagnosed when dementia precedes or occurs within 1 year of onset of motor Parkinsonian symptoms. However, if dementia develops more than a year after the onset of motor symptoms, this would be considered PDD.^2^

As well as shared symptomatology, both DLB and PDD are characterized by the presence of alpha-synuclein-containing Lewy bodies, and co-pathology with beta-amyloid and tau accumulation. A long-standing question is whether the two conditions have different underlying neurobiological processes.^3,4^ This has been challenging to address until recently, as neuropathological investigations are mostly restricted to end-stage disease. Neuroimaging has been limited, as conventional approaches mostly examined grey matter volume or thickness. Even in relatively large studies, these do not relate strongly to clinical symptoms^5^ and differences between DLB and PDD have been inconsistent. Some studies show greater atrophy in DLB than PDD;^6^ whilst others showed no differences in atrophy patterns between the two conditions.^7^

Instead, imaging techniques sensitive to changes in tissue composition, rather than volume and thickness, are likely to be more sensitive to any differences between DLB and PDD, and to relate to clinical severity.

One promising approach is quantitative susceptibility mapping (QSM), an MRI technique that calculates the magnetic susceptibility of brain tissue.^8,9^ This reflects brain tissue composition, including iron content, which is relevant in PDD and DLB, as excessive iron interacts with alpha-synuclein and triggers pathological aggregation^10^ and causes direct toxicity by generating free radicals.^11^ As well as brain tissue iron, differences in QSM values reflect changes in other metal ions such as calcium and copper, and variation in myelin content.^12^

We have previously shown that QSM values relate to cognitive severity in patients with Parkinson’s disease (PD) without dementia,^13^ and that regional QSM values predict future cognitive and motor severity in PD.^14^ There are no reports of direct DLB versus PDD comparisons using QSM, or between the combined grouping of Lewy body dementia compared with PD with normal cognition (PD-NC). However, in a study examining only the substantia nigra in DLB, higher QSM values were seen in DLB than in mild cognitive impairment with Lewy bodies (and compared with people with REM sleep behaviour disorder).^15^

Here, we used QSM to compare brain tissue composition between people with Lewy body dementia and those with PD-NC and between subtypes of Lewy body dementia: DLB and PDD. We predicted higher QSM values, which reflects more altered tissue composition, in Lewy body dementia than PD-NC, due to greater overall disease severity. We expected more pronounced alterations in DLB than PDD, due to higher levels of beta-amyloid pathology in DLB than PDD.^16^ Based on our previous findings in PD we also hypothesized that an increased QSM level would be associated with poorer cognitive and motor performance in all disease groups.

## Materials and methods

### Participants

Participants were recruited from PD and Lewy body dementia clinics at the National Hospital for Neurology and Neurosurgery, London, and affiliated clinics, between 2017 and 2023. Controls were recruited from patient’s spouses and university databases; 189 participants were included, comprising 86 people with PD who were cognitively unimpaired (PD-NC), 66 people with Lewy body dementia (45 DLB and 21 PDD) and 37 unaffected controls. Inclusion criteria were: a diagnosis of DLB, PDD or PD within 10 years of their respective diagnosis, aged 49–81 years. DLB diagnosis was made using DLB Consortium Criteria;^2^ PDD diagnosis according to Movement Disorder Society (MDS) PDD diagnostic criteria^17^ and PD diagnosis using Queen Square Brain Bank criteria; capacity to consent was a requirement for inclusion. Exclusion criteria were confounding neurological or psychiatric conditions and any contraindications to MRI. All participants were assessed by a neurologist (R.S.W.) to ascertain diagnosis of DLB, PDD or PD-NC and capacity to consent. All participants gave written, informed consent and the study was approved by the Queen Square Research Ethics Committee.

### Clinical assessments

All participants underwent detailed clinical assessments whilst on their usual medications, and levodopa equivalent daily dose was calculated.^18^ Disease-specific measures were obtained using validated questionnaires including the MDS Uniﬁed PD Rating Scale (MDS-UPDRS) that measures motor and non-motor domains.^19^ Part III of this scale was used to assess motor function, and total score was the summed score of parts I–IV.

Detailed neuropsychological assessment included the Montreal Cognitive Assessment (MoCA)^20^ as a global measure; and tests across each cognitive domain, as described previously.^21^ These test scores (Stroop colour, Hooper Visual Organization, word recognition, verbal fluency category and letter, MoCA) were also combined into a composite cognitive score, using the mean *Z*-score of each measure, as described previously.^21,22^

### MRI acquisition

Participants underwent MRI scanning on a Siemens Prisma-fit 3T MRI system using a 64-channel coil (Siemens Healthcare). T1-weighted magnetization-prepared 3D rapid, gradient-echo (MPRAGE) anatomical images were acquired with the following parameters: inversion time = 1100 ms, first echo time = 3.34 ms, repetition time (TR) = 2530 ms, flip angle = 7°, bandwidth = 200 Hz/pixel, resolution = 1 × 1 × 1 mm, matrix size = 256 × 256 × 176, acquisition time 6 min 3 s. Susceptibility-weighted images for QSM were acquired using a 3D flow-compensated spoiled gradient-recalled-echo sequence with the following parameters: TE = 18 ms, TR = 25 ms, flip angle = 12°, bandwidth = 110 Hz/pixel, resolution = 1 × 1 × 1 mm, matrix size = 204 × 224 × 160, acquisition time 5 min 41 s. Rate 2 × 1 parallel acquisition (GRAPPA) was enabled for both sequences.^23^

### QSM reconstruction

QSM reconstruction was performed as described previously.^14^ Briefly, QSM phase data were unwrapped using rapid opensource minimum spanning tree algorithm^24^ and brain masks calculated from magnitude images using the Brain Extraction Tool (BET2). Background field removal was performed using Laplacian boundary value extraction^25^ followed by 3D polynomial residual fitting.^26^ Multi-scale dipole inversion was used to generate susceptibility maps.^27^ All image processing was completed using MATLAB (The MathWorks, Inc., MA, USA). This pipeline has previously been optimized specifically for these acquisition parameters and disease cohort,^14,28^ and high-quality susceptibility maps for three representative subjects with LBD can be seen in Supplementary Fig. 1. A study-wide template was created from all participants’ T1-weighted images and QSM images were transformed into this space using advanced normalization tools (ANTs, http://stnava.github.io/ANTs/), as described previously.^13^

### Voxel-wise QSM analyses

Voxel-wise QSM analyses were performed throughout the brain using absolute QSM values, as has been performed previously.^13,14,29,30^ This was required because the application of a smoothing kernel, necessary in such voxel-wise analyses to account for coregistration inaccuracies and improve statistical conditioning, may collapse adjacent negative and positive values (commonly found in signed QSM data) to zero. The use of absolute QSM helps to avoid this issue, while also rendering values more comparable to the positive nature of R2*. Smoothing is not required for region of interest (ROI) analyses, so these were performed using the original signed QSM values. Images were spatially smoothed using a 3D Gaussian kernel [3 mm full-width-at-half-maximum (FWHM)] and then smoothing compensated.^30^ FSL Randomise was used to perform permutation analyses with threshold-free cluster enhancement, whereby 10 000 permutations were performed to identify significant clusters which were reported at family-wise error (FWE)-corrected *P* < 0.05 (*P*_FWE_ < 0.05) thresholds. Analyses were performed to compare differences between Lewy body dementia, PD-NC and controls, and between DLB and PDD, adjusting for age and sex. Secondary analyses separately compared DLB and PDD with both PD-NC and controls. Analyses were re-run with total brain volume (TBV) as an additional covariate to control for atrophy.^31,32^ TBV was defined as the total volume of grey- and white-matter fractions estimated by the SPM segment function. Further analyses were run to test associations between voxel-wise QSM smoothed absolute values and clinical measures (composite cognitive score, MoCA, UPDRS and UPDRS-motor), adjusting for age and sex. After analysis, the smoothed QSM template and statistical maps were transformed into MNI152 space (Montreal Neurological Institute, McGill University, Canada) for display purposes.^29^

### Region of interest QSM analyses

ROI analyses were performed to compare group differences in QSM in specific regions, and test associations between regional QSM and clinical measures. As the existing research using QSM in Lewy body dementia is limited to a single study,^15^ we selected regions in which QSM has been found to be related to cognitive and motor severity in PD,^13,14^ and Alzheimer’s disease.^33^ Our choice of ROIs was also informed by findings from studies using conventional measures of atrophy in Lewy body dementia^7,34-37^ as atrophy is a measure of neuronal loss^38^ likely to arise downstream of cellular iron dyshomeostasis.^10,39^ Specifically, we chose the nucleus basalis of Meynert (NBM), globus pallidus, caudate nucleus, putamen, substantia nigra pars reticulata (SNpr), substantia nigra pars compacta (SNpc), thalamus, hippocampus, insula, medial orbitofrontal, superior parietal and lateral occipital cortices; see the Supplementary material, ‘Methods’ section for details on ROI segmentation. Mean signed unsmoothed QSM values were calculated in bilateral ROIs. As the whole-brain voxel-wise analysis was performed using absolute QSM, the use of signed QSM for ROI analysis provided some indication of the contributions of diamagnetic and paramagnetic sources of susceptibility to our results. Interhemispheric differences were examined using *t*-tests; and then ROI means were averaged across hemispheres (see Supplementary Table 1A–C for differences between hemispheres).

We examined group differences in QSM values within ROIs using ANOVAs, adjusting for age and sex, and false discovery rate (FDR) was used to correct for multiple comparisons. We report fully corrected findings, and also uncorrected findings for completeness. Where significant F-statistics were observed, *post hoc* pairwise comparisons [corrected using Tukey’s honest significant difference (HSD)] were used to probe specific group differences. Where appropriate, median and interquartile range (IQR) of ROI mean QSM values are presented. Relationships between signed ROI mean QSM values and motor and cognitive severity were examined using linear regression, adjusting for age and sex, and FDR correcting across comparisons. ROI analyses were performed in R (v.4.2.2; https://www.r-project.org/). While we chose to employ standard few correction in our voxel-wise analyses due their extremely high dimensionality, we employed less conservative FDR correction for the much lower-dimensionality ROI analyses where we had prior expectation of involvement.

In order to compare our findings with those of Chen *et al*.,^15^ which is the only previous study to report QSM values in Lewy body dementia, we additionally extracted mean QSM values from the entire SN, combining SNpc and SNpr, and assessed between-study differences using *t*-tests.^15^

### Voxel-based morphometry

To compare the sensitivity of QSM to detect group differences with that of atrophy-based neuroimaging measures, and to further investigate the extent to which atrophy might act as a confound to QSM we performed voxel-based morphometry (VBM) analyses. SPM12 (http://www.fil.ion.ucl.ac.uk/spm/software/spm12) was used to segment T1-weighted images. A population template was derived for the whole study population using the diffeomorphic anatomical registration through exponentiated Lie algebra toolbox with a Gaussian smoothing kernel of 8 mm FWHM. Voxel-wise grey-matter probability maps were assessed using FSL Randomise with threshold-free cluster enhancement, and 10 000 permutations were performed to identify significant clusters at *P*_FWE_ < 0.05. Group comparisons were made using two-sample *t*-tests. Regression analyses were run to test the associations between voxel-wise grey matter probabilities and clinical measures including the composite cognitive score which takes into account detailed neuropsychological testing in five cognitive domains. All analyses were adjusted for age, sex and total intra-cranial volume. Significant clusters (extent threshold > 100 voxels) were labelled using the Harvard-Oxford Cortical Structural atlas.

### Additional statistical analyses

Demographic and clinical measures were compared between groups using two-tailed Welch’s *t*-tests or Mann–Whitney–Wilcoxon tests for non-normally distributed data. When three groups were compared, we used the Kruskal–Wallis test as the data were non-normally distributed, followed by the Dunn’s test for *post hoc* pairwise analysis with Bonferroni correction. We used linear regression to examine relationships between clinical variables. *P* < 0.05, Bonferroni corrected for multiple comparisons, was accepted as the threshold for statistical significance.

## Results

A total of 189 participants were included in our study: 66 Lewy body dementia (45 DLB; 21 PDD), 86 PD-NC and 37 controls. People with Lewy body dementia were older, had a higher proportion of males and were less educated than controls and PD-NC. Disease duration between Lewy body dementia and PD-NC did not differ. People with DLB and PDD did not differ in age, sex or education. There was no difference in dementia duration between DLB and PDD, but people with PDD had overall longer duration of disease due to PD prior to dementia diagnosis. As expected, people with Lewy body dementia had poorer cognition (MoCA and composite cognitive score) compared with PD-NC and controls. They also had increased overall disease severity (MDS-UPDRS total) and motor severity (MDS-UPDRS motor). We did not find significant differences in cognitive measures between PDD and DLB, but people with PDD had greater motor and overall disease severity than DLB. See Table 1 for demographic and clinical information.

**Table 1:** Demographics and clinical variables in Lewy body dementia, PD and controls

	Group					Statistical comparisons	
	DLB (*n* = 45)	PDD (*n* = 21)	LBD (*n* = 66)	PD-NC (*n* = 86)	Controls (*n* = 37)	DLB versus PDD	LBD versus PD-NC versus Controls
**Demographics**							
Age (years), mean (SD)	72.6 (5.6)	70.5 (6.5)	71.9 (6.0)	63.6 (7.9)	65.8 (9.0)	W = 580.5 *P* = 0.14	**χ^2^ = 39.15** **df = 2 *P* = 3.16 × 10^−9a,b^**
Sex, F/M	4/41	4/17	8/58	41/45	17/20	χ**^2^** = 0.60 df = 1 *P* = 0.44	**χ** ^2^ **= 23.23** **df = 2** ***P* = 9.03 × 10^−6a,b^**
Diagnosis (years), mean (SD)	2.2 (1.9)	8.7 (5.5)	4.3 (4.6)	4.2 (2.5)	−	**W = 77** ***P* = 3.90 × 10^−8^**	W = 3288 *P* = 0.092
Dementia (years), mean (SD)	2.2 (1.9)	1.9 (1.8)	2.1 (1.8)	–	–	W = 416.5 *P* = 0.43	–
Education (years), mean (SD)	15.9 (3.3)	16.0 (3.6)	15.9 (3.4)	17.2 (2.8)	17.5 (2.5)	W = 471.5 *P* = 0.99	**χ^2^ = 6.80** **df = 2** ***P* = 0.03^a,b^**
Clinical features							
MoCA, mean (SD)	21.2 (5.4)	22.3 (3.9)	21.5 (5.0)	28.1 (1.9)	28.6 (1.6)	W = 432 *P* = 0.58	**χ^2^ = 99.06** **df = 2** ***P* < 2.20 × 10^−16a,b^**
Composite cognitive score, mean (SD)	−2.78 (1.77)	−2.33 (1.50)	−2.65 (1.70)	−0.20 (0.73)	0.00 (0.56)	W = 348 *P* = 0.39	**χ^2^ = 102.13** **df = 2** ***P* < 2.20 × 10^−16a,b^**
MDS-UPDRS, mean (SD)	65.8 (30.4)	83.1 (23.5)	71.3 (29.4)	44.2 (21.3)	8.3 (5.1)	**W = 296.5** ***P* = 0.016**	**χ^2^ = 112.92** **df = 2** ***P* < 2.20 × 10^−16a,b,c^**
MDS-UPDRS-motor, mean (SD)	33.8 (17.6)	40.6 (11.1)	36.0 (16.1)	20.4 (14.2)	4.8 (4.3)	**W = 327** ***P* = 0.046**	**χ^2^ = 94.20** **df = 2,** ***P* < 2.20 × 10^−16a,b,c^**
LEDD	263.89 (252.63)	773.33 (374.30)	425.98 (378.7)	472.55 (252.88)	–	**W = 113** ***P* = 5.56 × 10^−7^**	W = 2360 *P* = 0.07
AChEIs medication, yes/no	38/7	8/13	46/20	–	–	**χ^2^ = 12.45** **df = 1** ***P* = 0.00042**	–

Significant group differences highlighted in bold text. AChEIs = acetylcholinesterase inhibitor; DLB = dementia with Lewy bodies; LBD = Lewy body dementia; LEDD = total levodopa equivalent dose; MDS-UPDRS = Movement Disorders Society Unified Parkinson’s Disease Rating Scale; MoCA = Montreal Cognitive Assessment; PD = Parkinson's disease; PDD = Parkinson’s disease dementia; PD-NC = Parkinson’s disease with normal cognition; SD = standard deviation.

^a^Significant differences in *post hoc* comparisons between Controls and LBD.

^b^Significant differences in *post hoc* comparisons between PD-NC and LBD.

^c^Significant differences in *post hoc* comparisons between Controls and PD-NC.

### Voxel-wise QSM in Lewy body dementia and between Lewy body dementia subtypes

**Figure 1:**
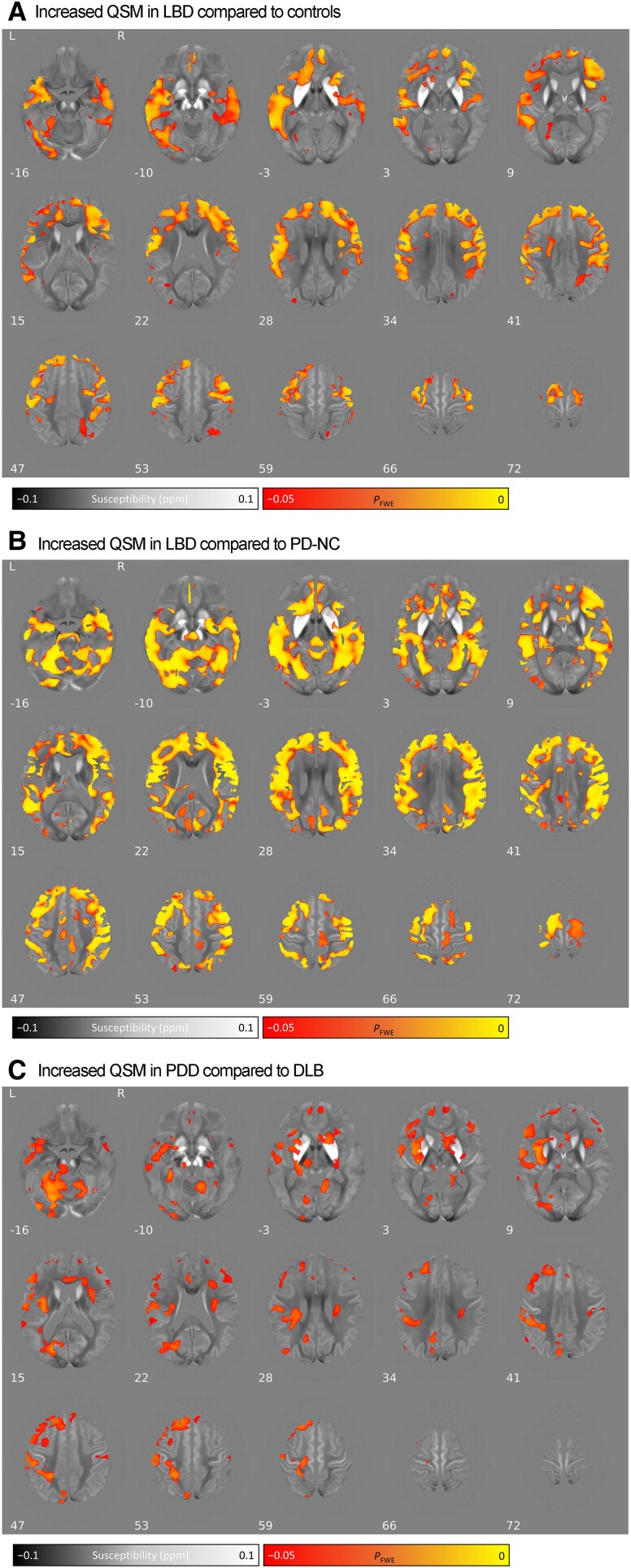
**Voxel-wise group comparison of absolute QSM values throughout the brain.** (**A**) LBD compared with controls. (**B**) LBD compared with PD-NC. (**C**) PDD compared with DLB. Results are overlaid on the study-wide QSM template in MNI152 space, and numbers represent axial slice location in MNI152 space. Left side is shown on the *left*. Red/yellow clusters represent voxels where a significant relationship was seen at FWE-corrected *P* < 0.05, with increased absolute QSM values. DLB = dementia with Lewy bodies; FWE = family-wise error; LBD = Lewy body dementia; MNI = Montreal Neurological Institute; PDD = Parkinson’s disease dementia; PD-NC = Parkinson’s disease with normal cognition; QSM = quantitative susceptibility mapping.

We found increased absolute QSM values in several brain regions in Lewy body dementia compared with controls and PD-NC (*P*_FWE_ < 0.05). For both comparisons, the bilateral superior frontal, bilateral medial temporal, left superior temporal, right precentral and left cerebellar cortex were involved (Fig. 1A and B). There were no brain regions with increased absolute QSM values in controls and PD-NC compared with Lewy body dementia.

For PDD compared with DLB, we found widespread increased QSM values, with differences in the bilateral cerebellar, hippocampi, right amygdala, left SNpr, left frontal, left precentral, left paracentral and left precuneus (*P*_FWE_ < 0.05) (Fig. 1C). There were no brain regions in which the reverse relationship was observed. See Supplementary Fig. 2 for comparisons between PDD and controls, PDD and PD-NC, DLB versus controls and DLB versus PD-NC.

**Figure 2:**
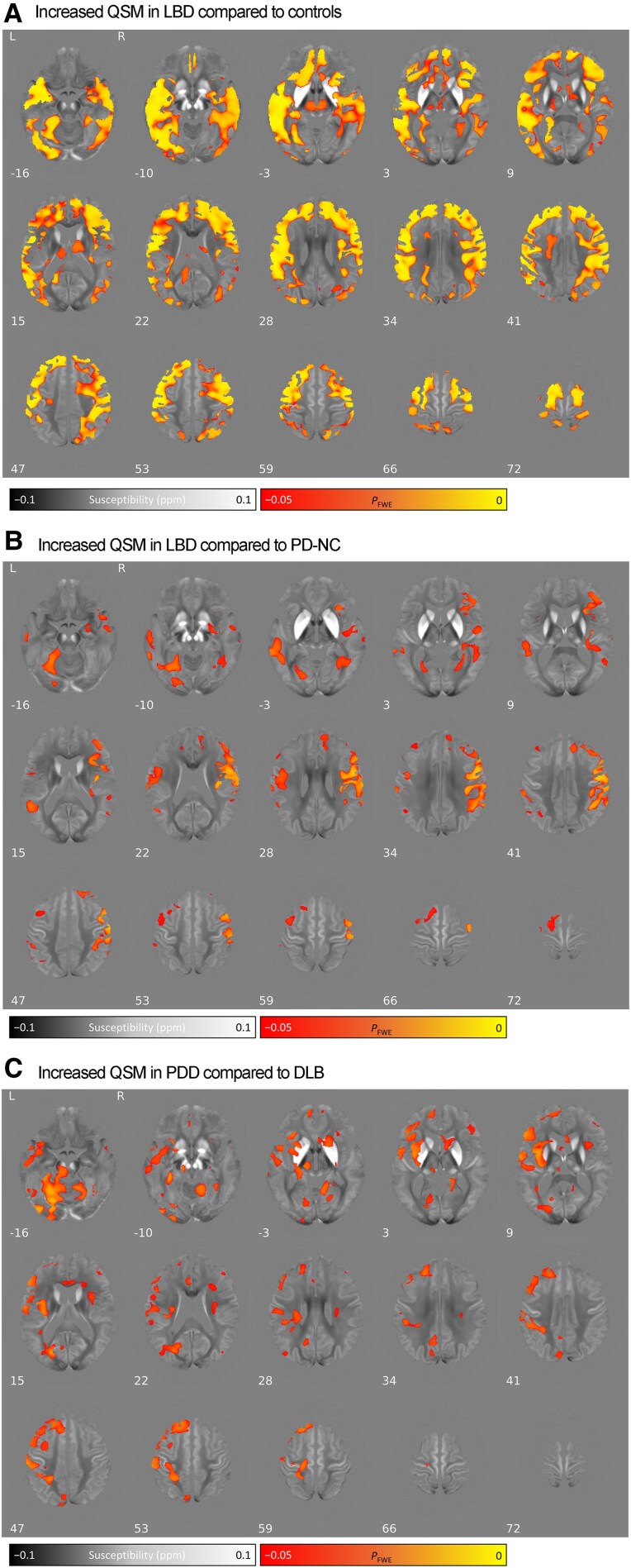
**Group comparison of absolute QSM values in whole brain analysis, additionally corrected for TBV.** (**A**) Increased QSM in LBD compared with controls. (**B**) Increased QSM in LBD compared with PD-NC. (**C**) Increased QSM in PDD compared with DLB. Results are overlaid on the study-wise QSM template in MNI152 space, and numbers represent axial slice location in MNI152 space. Left side is shown on the left. Red/yellow clusters represent voxels where a significant relationship was seen at FWE-corrected *P* < 0.05 (corrected for age, sex and TBV). DLB = dementia with Lewy bodies; FWE = family-wise error; LBD = Lewy body dementia; MNI = Montreal Neurological Institute; PDD = Parkinson’s disease dementia; PD-NC = Parkinson’s disease with normal cognition; QSM = quantitative susceptibility mapping; TBV = total brain volume.

We performed a secondary analysis correcting for atrophy using total brain volume (TBV), to test whether this was driving the observed group differences. We still observed increases in QSM values in Lewy body dementia and in PDD, even when using TBV correction (Fig. 2 and Supplementary Fig. 3). There were greater differences between LBD and controls but less for LBD compared with PD-NC and PDD compared with DLB.

### Relationships between voxel-wise QSM and clinical measures in Lewy body dementia

**Figure 3:**
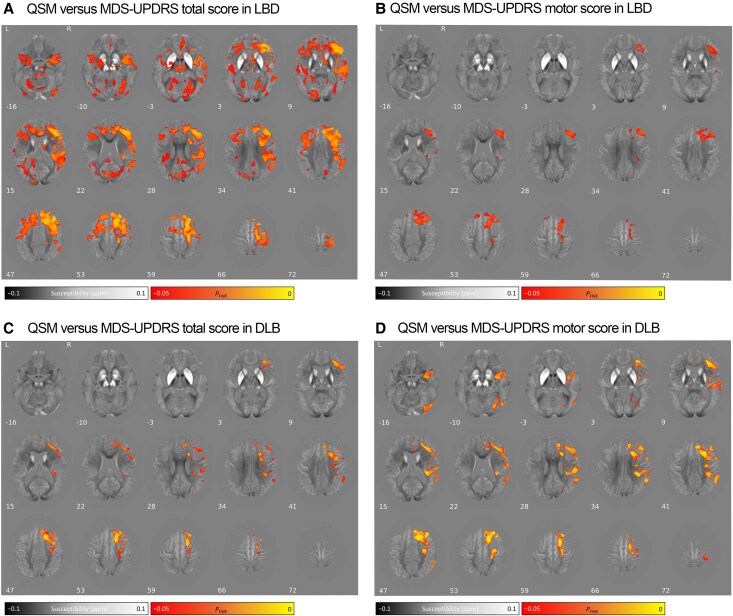
**Voxel-wise association of absolute QSM values with MDS-UPDRS scores in people with Lewy body dementia.** Association between absolute QSM values and: (**A**) overall disease severity (MDS-UPDRS total) in LBD; (**B**) motor severity (MDS-UPDRS III) in LBD; (**C**) overall disease severity (MDS-UPDRS total) in DLB; (**D**) motor severity (MDS-UPDRS III) in DLB. Results are overlaid on the study-wide QSM template in MNI152 space, and numbers represent axial slice location in MNI152 space. Left side is shown on the *left.* Red/yellow clusters represent voxels where a significant positive relationship was seen at *P*_FWE_ < 0.05. DLB = dementia with Lewy bodies; FWE = family-wise error; LBD = Lewy body dementia; MDS-UPDRS = Movement Disorder Society Unified Parkinson’s Disease Rating Scale; MNI = Montreal Neurological Institute; QSM = quantitative susceptibility mapping.

We found a significant positive correlation between absolute QSM values and disease severity, measured using total MDS-UPDRS, in several brain areas in people with Lewy body dementia. This means that higher absolute QSM values were found in subjects with greater MDS-UPDRS scores. These correlations were found in areas including the right thalamus, left pallidum, bilateral SNpr and SNpc, bilateral middle frontal, temporal and lateral occipital lobes, right precentral and superior frontal cortices (Fig. 3A; *P*_FWE_ < 0.05). We also found significant positive associations between absolute QSM values and motor severity in the bilateral superior frontal, right insula and right middle frontal lobes (Fig. 3B; *P*_FWE_ < 0.05). These relationships were also found in DLB (Fig. 3C and D; *P*_FWE_ < 0.05), but not in PDD. We did not find significant voxel-wise associations between absolute QSM values and cognitive measures in any group.

### ROI QSM differences in Parkinson's disease and Lewy body dementia subtypes

**Figure 4:**
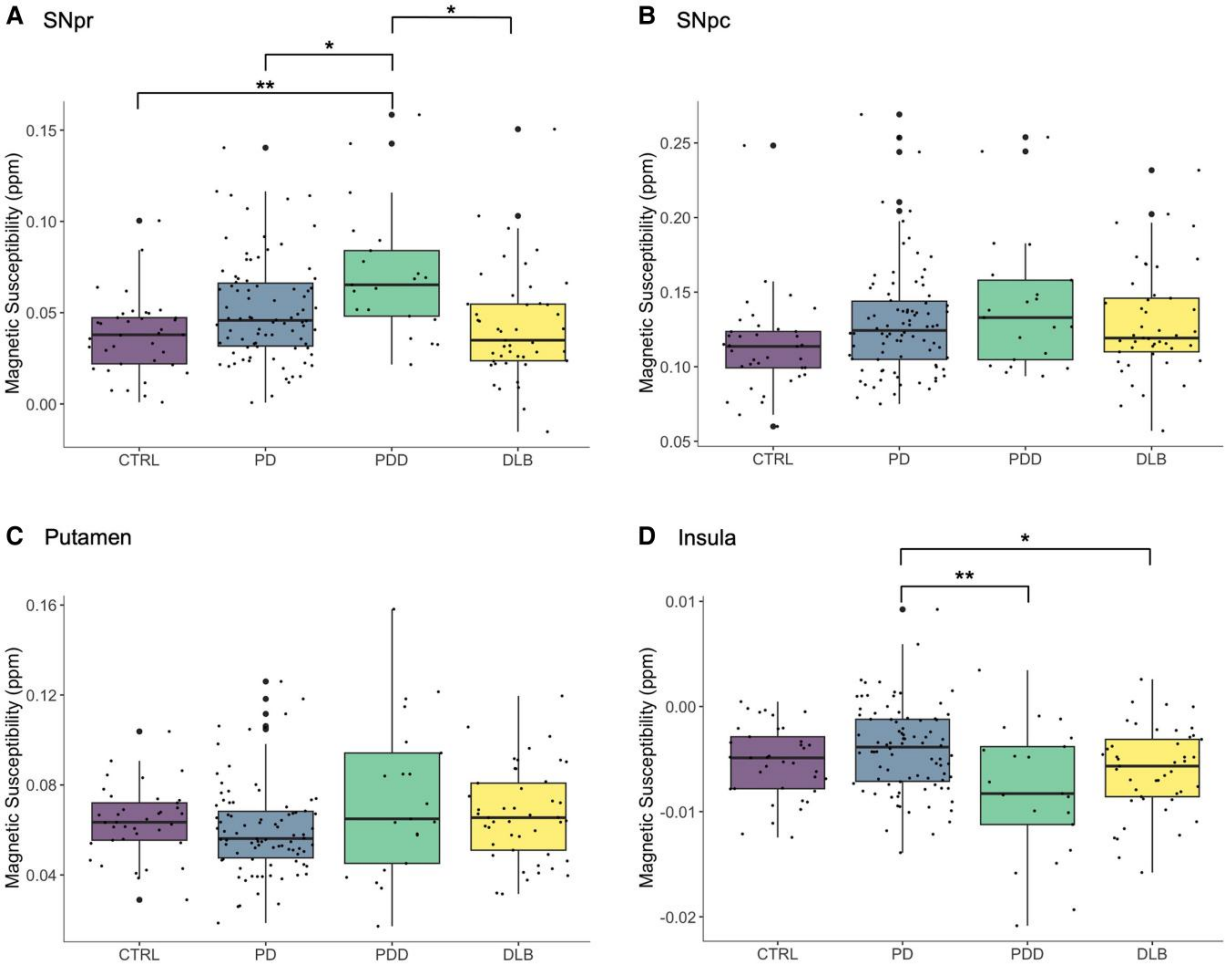
**Signed QSM values in regions of interest showing group-level differences.** QSM values for LBD subtypes and people with Parkinson’s in: (**A**) substantia nigra pars reticulata (SNpr); (**B**) QSM values in the substantia nigra pars compacta (SNpc); (**C**) QSM values in the putamen; (**D**) QSM values in the insula. For each ROI, we show the individual’s mean QSM values, which have been averaged across both hemispheres. The horizontal bar is the group median and the box indicates the interquartile range between the first (*bottom*) and third (*top*) quartiles. Note that QSM values for ROI analyses are signed, rather than absolute values (see ‘Materials and methods’ section). ANOVAs were performed to identify group level differences. *Post hoc* pairwise comparisons for significant regions: *P < 0.05, **P < 0.001. DLB = dementia with Lewy bodies; LBD = Lewy body dementia; PDD = Parkinson’s disease dementia; QSM = quantitative susceptibility mapping; ROI = region of interest.

In ROI analyses we found group differences in QSM values in four regions, but only the SNpr and insular survived corrections for multiple comparisons (see Fig. 4). Specifically, for the SNpr, we found increased QSM values when comparing PDD to controls [PDD median = 0.065 (IQR = 0.036); controls median = 0.038 (IQR = 0.025); Tukey *P*_HSD_ = 0.00015]. We similarly found increases comparing PDD to PD-NC [PD-NC median = 0.046 (IQR = 0.034), *P*_HSD_ = 0.029] and PDD compared with DLB [DLB median = 0.035 (IQR = 0.031); *P*_HSD_ = 0.0011] (Fig. 4A and see Table 2 for comparisons). ANOVA revealed group differences in the SNpc (Fig. 4B and Table 2) and putamen (Fig. 4C and Table 2) but these did not survive correction for multiple corrections.

**Table 2:** Regional mean signed magnetic susceptibilities

ROI	DLB (*n* = 45)	PDD (*n* = 21)	PD-NC (*n* = 86)	Controls (*n* = 37)	F-statistic	Uncorrected *P*	*P*_FDR_
NBM	0.14 (0.049)	0.16 (0.076)	0.14 (0.035)	0.15 (0.044)	2.14	0.10	0.20
Globus pallidus	0.10 (0.023)	0.11 (0.029)	0.11 (0.020)	0.10 (0.022)	1.69	0.17	0.28
Caudate	0.043 (0.022)	0.046 (0.030)	0.041 (0.017)	0.043 (0.013)	1.54	0.21	0.28
Putamen	0.065 (0.030)	0.065 (0.049)	0.056 (0.021)	0.063 (0.017)	2.73	0.045	0.14
**SNPr**	**0.035** (**0.031)**	**0.065** (**0.036)**	**0.046** (**0.034)**	**0.038** (**0.025)**	**7**.**25**	**0**.**00013**	**0.0016^a,b,c^**
SNPc	0.12 (0.036)	0.13 (0.053)	0.12 (0.040)	0.11 (0.024)	2.96	0.034	0.14^b^
Thalamus	−0.0070 (0.0089)	−0.0045 (0.0043)	−0.0044 (0.0062)	−0.0031 (0.0049)	0.42	0.74	0.74
Hippocampus	−0.0082 (0.0056)	−0.0081 (0.10)	−0.0071 (0.0074)	−0.0092 (0.0079)	0.82	0.48	0.52
**Insula**	**−0.0061** (**0.0041)**	**−0.0081** (**0.0063)**	**−0.0041** (**0.0046)**	**−0.0053** (**0.0034)**	**6**.**01**	**0**.**00063**	**0.0038^b,d^**
Medial orbitofrontal	−0.0054 (0.0094)	−0.0081 (0.0060)	−0.0043 (0.0067)	−0.0068 (0.0084)	1.02	0.39	0.47
Superior parietal	0.0027 (0.0050)	0.00085 (0.0051)	0.0017 (0.0046)	−0.00016 (0.0057)	2.55	0.058	0.14
Lateral occipital	−0.0019 (0.0066)	−0.0048 (0.0063)	−0.0022 (0.0060)	−0.0010 (0.0076)	1.54	0.21	0.28

Median (IQR) of regional mean signed magnetic susceptibility (in ppm) by group. For the globus pallidus, insula and lateral occipital ROIs, mean (standard deviation) is presented as the mean QSM values were normally distributed in these regions. Normality of regional mean signed magnetic susceptibility (in ppm) were tested using the Shapiro–Wilk test. Group level comparison with ANOVAs are shown, FDR corrected for multiple comparisons. Bold signifies group differences that survived FDR correction for multiple comparisons. DLB = dementia with Lewy bodies; FDR = false discovery rate; IQR = interquartile range; PDD = Parkinson’s disease dementia; LBD = Lewy body dementia; PD-NC = Parkinson’s disease with normal cognition; NBM = nucleus basalis of Meynert; QSM = quantitative susceptibility mapping; ROI = region of interest; SNpr = substantia nigra pars reticulata; SNpc = substantia nigra pars compacta.

^a^ *Post hoc* pairwise comparisons for significant regions Controls and PDD.

^b^ *Post hoc* pairwise comparisons for significant regions PD and PDD.

^c^ *Post hoc* pairwise comparisons for significant regions PDD and DLB.

^d^ *Post hoc* pairwise comparisons for significant regions PD and DLB.

In the insula, we found significantly higher QSM values for PD-NC compared with PDD [PD-NC mean = −0.0041 (SD = 0.0046), PDD mean = 0.0041 (SD = 0.0063); diff = 0.0040; Tukey *P*_HSD_ = 0.00070]; and for PD-NC compared with DLB [DLB mean = 0.0061 (SD = 0.0041); diff = 0.0020; Tukey *P*_HSD_ = 0.048] (Fig. 4D and Table 2).

### Relationships between ROI QSM values and clinical measures

**Figure 5:**
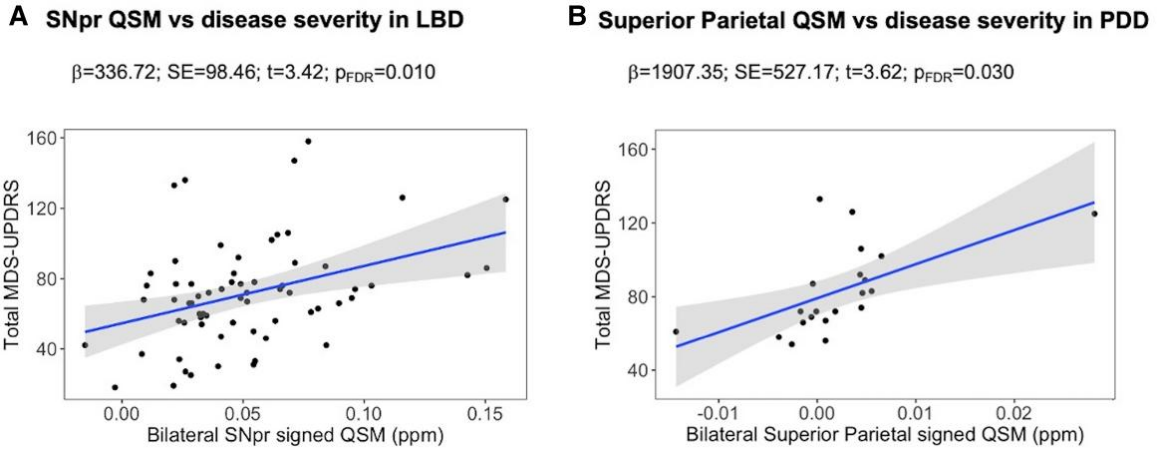
**Relationships between QSM values and disease severity in regions of interest.** ROI mean QSM values significantly associated with disease severity (measured using total MDS-UPDRS score) in: (**A**) substantia nigra pars reticulata (SNpr) in LBD; (**B**) superior parietal cortex in PDD. Signed values are used in this analysis and results are adjusted for age and sex, with false discovery rate-corrected *P*-values presented. LBD = Lewy body dementia; PDD = Parkinson’s disease dementia; QSM = quantitative susceptibility mapping; ROI = region of interest; SNpr = substantia nigra pars reticulata; total MDS-UPDRS = Movement Disorder Society Unified Parkinson’s Disease Rating Scale total score.

We found a significant positive association between overall disease severity (MDS-UPDRS total score) and ROI mean signed QSM values in the SNpr in Lewy body dementia (β = 336.72; *P*_FDR_ = 0.010) (Fig. 5A). In this group, we also found a positive association of motor severity with ROI mean signed QSM values in the SNpr but this was not significant (β = 159.45; *P*_FDR_ = 0.08). In people with PDD, disease severity was also positively associated with QSM values, but only in the superior parietal cortex (β = 1907.35; *P*_FDR_ = 0.03) (Fig. 5B). Two PDD participants had mean QSM values that were outlying, −0.014 (which is <median – 1.5 × IQR) and 0.028 (which is >median + 1.5 × IQR), respectively. We re-ran the regression analysis excluding these participants and found an even stronger relationship between higher QSM values and greater disease severity [β = 4431.69; standard error (SE) = 1467.59; *t* = 3.02; *P* = 0.0086]. We did not find any other significant associations between clinical measures and regional mean signed QSM values in Lewy body dementia, PDD or DLB (see Supplementary Table 2 for ROI associations with clinical measures in Lewy body dementia, Supplementary Table 3 for DLB and Supplementary Table 4 for PDD).

### Comparison of Lewy body dementia QSM values with previously reported values

Our results did not differ from the previously reported QSM study in Lewy body dementia.^15^ We found that QSM values in the whole SN aligned with one another across studies, and found no between-study group differences after correction for multiple comparisons (see Supplementary Tables 5 and 6).

### Voxel-based morphometry

VBM comparing Lewy body dementia with controls revealed significant volume loss in Lewy body dementia in the left parahippocampal gyrus and middle frontal gyrus, and in the right inferior temporal gyrus, precentral gyrus and precuneus (*P*_FWE_ < 0.05). We also found volume loss in the bilateral parahippocampal gyrus frontal, bilateral inferior temporal gyrus and left temporal pole in DLB compared with controls, right precentral gyrus, left middle frontal gyrus and right intracalcarine sulcus and in the right superior frontal cortex for PDD compared with controls (*P*_FWE_ < 0.05). We found volume loss in Lewy body dementia relative to PD-NC in the bilateral parahippocampal gyri, left inferior temporal gyrus and temporal pole, and right insular cortex (*P*_FWE_ < 0.05). We also found volume loss in the left parahippocampal gyrus in DLB relative to PD-NC, and in the bilateral cerebellum, left parietal operculum cortex and right precentral gyrus in PDD relative to PD-NC (*P*_FWE_ < 0.05). We did not find any significant VBM differences between people with DLB and PDD. See Supplementary Table 7 for the full list of regional VBM group differences.

We found widespread associations between atrophy and cognitive severity in Lewy body dementia, we found increasing atrophy with increasing cognitive severity in the bilateral cerebellum and cingulate gyri, left middle and superior temporal gyri, left precentral gyrus, as well as the right angular and supramarginal gyri and right lateral occipital cortex (*P*_FWE_ < 0.05). We also found atrophy increasing with increasing disease severity score (MDS-UPDRS) in the bilateral cerebellum and left occipital fusiform gyrus (*P*_FWE_ < 0.05). In PDD, we found no associations with MDS-UPDRS, although we did find atrophy increasing with increasing cognitive severity in the bilateral temporal fusiform cortex and superior parietal lobules, left angular, supramarginal, precentral and middle temporal gyri, left central opercular cortex, right frontal orbital and paracingulate cortices and right temporal pole (*P*_FWE_ < 0.05). Lastly, in DLB we found increasing atrophy with increasing cognitive severity in the right cerebellum, although no associations with disease severity (*P*_FWE_ < 0.05). See Supplementary Table 8 for the full list of regional VBM regression analyses.

## Discussion

We used QSM to examine differences in brain magnetic susceptibility in Lewy body dementia and between Lewy body dementia subtypes. We found significant differences in QSM values between Lewy body dementia and both PD-NC and controls using voxel-wise analysis throughout the brain. When comparing Lewy body dementia subtypes, we found widespread increased QSM values in PDD compared with DLB. These differences remained after correcting for atrophy, and we did not find similar differences between these groups when using a more conventional approach comparing brain volumes using VBM. We also showed increased QSM values associated with greater disease severity in several cortical regions in Lewy body dementia, suggesting QSM may have potential as a neuroimaging marker of disease activity in Lewy body dementia.

Our finding of widespread altered cortical QSM values in Lewy body dementia is consistent with neuropathological findings of widespread cortical Lewy-related accumulations and Alzheimer’s pathology.^4,40,41^ Although cortical atrophy has been previously detected in Lewy body dementia using measures of volume or cortical thickness, these are generally not consistent across studies.^42-44^ The widespread QSM increases in Lewy body dementia versus other groups seen here suggest that neuroimaging measures sensitive to changes in tissue composition and microstructure, rather than differences in volume or thickness, are likely to be more effective measures in distinguishing between Lewy body dementia subtypes.

We also showed clear positive relationships between QSM values and overall disease severity, measured using the MDS-UPDRS total score, though QSM values did not relate to cognitive performance. Conversely, while we did not find widespread atrophy associated with overall disease severity, we did find widespread associations between atrophy and cognitive severity in Lewy body dementia, as measured by VBM. Previous work examining QSM in PD, including our own,^13,14^ has shown a relationship between QSM values and cognition as well as motor severity in PD. However, we previously did not find concurrent associations between atrophy and cognition in PD.^13,14^ There are several possible reasons for the lack of association between QSM and cognition in Lewy body dementia. Previous work showing this relationship was only at earlier stages, in PD without dementia, and higher QSM values may be a better indicator of early changes relating to cognitive impairment. In Lewy body dementia, where patients have established dementia, additional changes in cognition may be better explained by measures other than QSM. There is also a higher likelihood of co-pathology at later disease stages.^45^ It is not yet clear how the combination of tissue changes at this later disease stage contributes to the single bulk magnetic susceptibility measurement provided per voxel by QSM. Additional measures may help to disambiguate these tissue changes.^46,47^

While increased iron bound in ferritin macromolecules is the greatest contributor to paramagnetic tissue susceptibility,^48,49^ concurrent increases in the diamagnetic susceptibility contribution in the same tissues may result in no net increase in susceptibility measured using QSM. For example, increases in diamagnetic metals such as copper, magnesium and calcium have also been observed in Lewy body diseases,^50,51^ while pathological proteins would also be expected to have a diamagnetic susceptibility contribution as they contain a high number of paired electrons,^52,53^ and together, this could lower the overall QSM value, even in a voxel with an increased ferritin level. Further, neurotoxic reactive oxygen species produced by excess tissue iron may degrade ferritin,^54^ which could decrease the measured susceptibility value. Susceptibility has also been shown to be sensitive to microstructural changes in orientation.^55^ The observation that QSM values related strongly to overall disease severity, does, however, indicate that it has relevance as a measure of tissue involvement. These results also suggest that, while QSM is more strongly associated with cognitive severity than atrophy in early-stage PD,^13,14^ atrophy is a better biomarker for cognitive severity in late-stage disease than QSM.

Previous studies have found grey matter reductions in posterior and parietal areas for patients with PDD and DLB.^56^ Similarly, we found reductions in volume in the right precuneus. Additionally, we found a correlation between atrophy changes and cognitive severity in our VBM analysis. However, we did not find susceptibility changes in posterior and parietal areas in our QSM analysis in LBD, or any relating to cognitive severity. It is possible that QSM detects different processes than those detected by grey matter atrophy, and that this reflects the difference in the regional involvement seen between the two forms of analysis. It is also possible that the atrophy itself affects the QSM values, in a more complex way than can be accounted for by TBV correction.^32^ Future work could explicitly investigate the relationship between grey matter atrophy and QSM values in more detail.

We found more widespread cortical QSM value increases in PDD than DLB, even after correcting for atrophy. This was counter to our prediction that people with DLB would show higher QSM values than PDD, based on studies showing higher levels of beta-amyloid accumulation in DLB than PDD.^16^ This finding may be due to a sampling effect, of particular differences in either our PDD or DLB cohort, and will need to be replicated in studies with larger patient numbers. The lower QSM values in DLB could also reflect differences in the cortical protein accumulations in DLB compared with PDD with differing diamagnetic properties.^52,53^

Our ROI analysis showed an increase in QSM values in the SNpr in PDD compared with PD-NC, and even higher QSM values in PDD compared with controls, as well as a positive relationship between QSM values and disease severity in the SNpr. This is consistent with previous work showing increased QSM values in SN in PD^57^ and with disease severity.^14^ However, our ROI analysis, which used signed, as opposed to absolute QSM values, did not show the diffuse cortical changes in Lewy body dementia compared with controls or PD that we had shown in our voxel-wise QSM analysis. This difference is likely to arise from the contribution of both paramagnetic and diamagnetic sources to the absolute QSM values in our voxel-wise analysis.^14^ Iron bound to ferritin is the key contributor to magnetic susceptibility and is paramagnetic.^48^ However, there are several diamagnetic contributions to the overall susceptibility and QSM values. These include metals such as calcium, magnesium and copper in some oxidation states.^49^ Diamagnetic sources also include myelin,^58^ which may be increased in some cortical regions in PD.^59^ Pathological proteins such as alpha-synuclein, beta-amyloid and tau are also likely to be diamagnetic.^52,53^

There are some limitations to consider in this work. The QSM values reported here reflect bulk magnetic susceptibilities with contributions from both paramagnetic and diamagnetic sources. It is possible for these different sources to be disambiguated, if data with a sufficient number of echoes (and a quantitative T2 map) are acquired.^46,47^ This would then uncover the underlying sources by calculation of separate paramagnetic and diamagnetic susceptibility maps. However, this was not possible with our current dataset, and such approaches still require validation in large cohorts.

Our control group had not been prospectively collected, resulting in differences in age and sex between PD-NC and controls with Lewy body dementia. Although we had corrected for both of these factors in our analyses, it would be optimal to have demographically matched groups, especially considering that age contributes to QSM values.^60^

Similarly, although people with PDD and DLB were matched in terms of duration of dementia, people with PDD had a longer overall disease duration, as dementia can emerge several years after a diagnosis of PD. Given the nature of PDD and DLB diagnostic criteria, patients can either be matched by overall PD duration or dementia duration. We matched for dementia duration given that this more closely relates to the patient’s clinical condition, and was more relevant to the comparison being conducted here between DLB and PDD. Future work could examine patients matched for overall Lewy body disease duration but this would remain challenging given that the association of disease duration with neuropathological^61^ and clinical severity^62^ is complex, with some patients having shorter disease duration but a more severe clinical phenotype.

Our PDD sample, in particular, is small due to challenges in recruitment. We were able to find significant group-level differences in voxel-wise and regional QSM values suggesting that the study was adequately powered in this regard. However, the PDD was too small to detect significant correlations between QSM values (both voxel-wise and regional) and disease severity measures.

Regarding our QSM acquisition, the consensus recommendation is to acquire multi-echo as opposed to single-echo data,^26^ as this allows for a more precise calculation of the underlying field shift, ΔB_0,_^63^ and therefore yields more accurate susceptibility maps. Additionally, it should be noted that some structures where we report findings, notably mesial temporal structures and insular cortex, can be particularly vulnerable to non-local susceptibility artefacts due to adjacent bone, air and vasculature. Although we are confident that our optimized QSM pipeline and quality control protocol minimize these effects (see Supplementary Fig. 1), our results should be interpreted with this in mind.

Finally, our findings are based on group-level analyses, but to be applicable in clinical practice, we will require information based on QSM measurements at an individual level.

## Conclusion

In summary, we show that QSM values are higher in Lewy body dementia than PD with normal cognition and higher in PDD compared with DLB. We found that QSM values increased with worse overall disease severity. These findings were seen in cortical areas, and also in the SNpr. Our observations suggest distinct underlying neurobiology in PDD compared with DLB, and demonstrate that QSM can detect brain changes relating to real-world clinical measures of disease severity in Lewy body dementia. Future work, applying susceptibility source separation methods to multi-echo gradient echo MRI data may show even greater sensitivity to clinical severity in Lewy body dementia.

## Supplementary Material

awaf325_Supplementary_Data

## Data Availability

Imaging and clinical data used in this study will be shared upon reasonable request to the corresponding author. All data and statistics generated from this study are presented in the manuscript and Supplementary data.
