# Supplementary material for: Quantitative susceptibility mapping reveals differences between subtypes of Lewy body dementia
Source: Brain. 2025 Sep 4;149(3):1058–69. doi: 10.1093/brain/awaf325 (PMC13017651; doi:10.1093/brain/awaf325)
Supplement: awaf325_Supplementary_Data [file awaf325_supplementary_data.docx]

# Title

Quantitative Susceptibility Mapping reveals differences between subtypes of Lewy body dementia

# Authors

Bhome R,^1,2^ Thomas GEC,^1^ Hannaway N^1^, Dobreva I^1^, Zarkali A^1^, Shmueli K,^3^ Cole JH,^1,2^ Weil RS,1,4,5

1. Dementia Research Centre, University College London, 8-11 Queen Square, London,

WC1N 3AR, UK

1. UCL Hawkes Institute, University College London, London, WC1V 6LJ, UK
2. Department of Medical Physics and Biomedical Engineering, UCL, London, UK
3. Wellcome Centre for Human Neuroimaging, University College London, 12 Queen

Square, London, WC1N 3AR

1. Movement Disorders Consortium, National Hospital for Neurology and Neurosurgery,

Queen Square, London, WC1N 3AR

# Sample Size calculation

For QSM measures in Lewy body disease, our previous work^1^ found significant differences in hippocampal QSM between Parkinson’s groups with higher and lower risk of progression to dementia, with Cohen’s d =0.72 (mean in lower risk group 0.0164 (SD 0.0024), mean in higher risk group 0.0181 (SD 0.0023). A total sample of 64 LBD participants (32 DLB and

32 PDD) gives 80% power to detect group differences. Of note, the only previous work using QSM in Lewy body dementia^2^ found significant differences in substantia nigra QSM levels between 36 people with DLB and 15 people with mild cognitive impairment with Lewy bodies (MCI-LB).

Due to recruitment challenges, we recruited 21 PDD and 45 DLB participants, providing a total of 66 people with Lewy body dementia. While this is lower than planned for in the PDD group, we were able to find significant differences as presented in the results, suggesting that we had sufficient power.


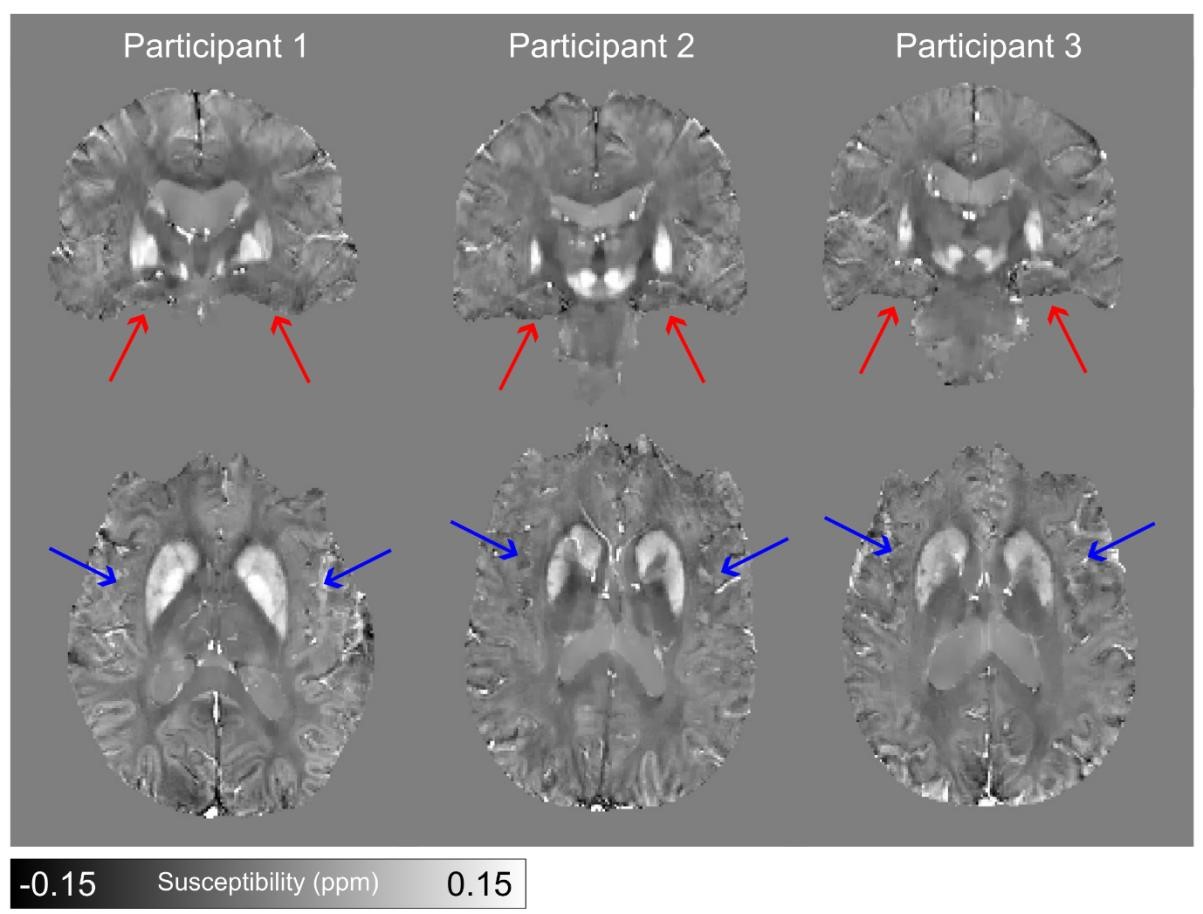


**Supplementary Figure 1. Example subject** **-level susceptibility maps.** Example maps for 3 participants with Lewy body dementia are shown. Red arrows indicate mesial temporal structures, while blue arrows indicate insular cortices. Both these structures can be affected by non-local susceptibility artefacts emanating from bone, CSF and vasculature. As can be seen, our preprocessing pipeline performed well including in these regions.

# Region of Interest Extraction

FSL-FIRST was used to automatically segment the Globus Pallidus, Caudate Nucleus,

Putamen, Thalamus and Hippocampus on the study-wise MPRAGE template. The Substantia Nigra was segmented from the QSM and MPRAGE templates using a QSM segmentation tool^3^, publicly available via [*www.mricloud.org.*](http://www.mricloud.org/) The Substantia Nigra was further manually subdivided into the SNPr and SNPc on the MPRAGE template, guided by the T1 shortening effects exhibited by the neuromelanin-rich neurons of the SNpc compared to the SNpr.

The Insula, medial orbitofrontal, superior parietal and lateral occipital regions were defined using the Desikan-Killiany-Tourville digital atlas^4^. The OASIS-30 template and OASIS-

TRT-20 joint fusion atlas were obtained from Mindboggle’s repository

[(http://www.mindboggle.info/data)](http://www.mindboggle.info/data). The study-wise template to OASIS-30 space non-linear transformations were calculated by deformable b-spline co-registration in ANTs. The cortical regions of interest were intersected with a study-wise average grey matter mask and binarised at a grey matter density cut-off of 0.5.


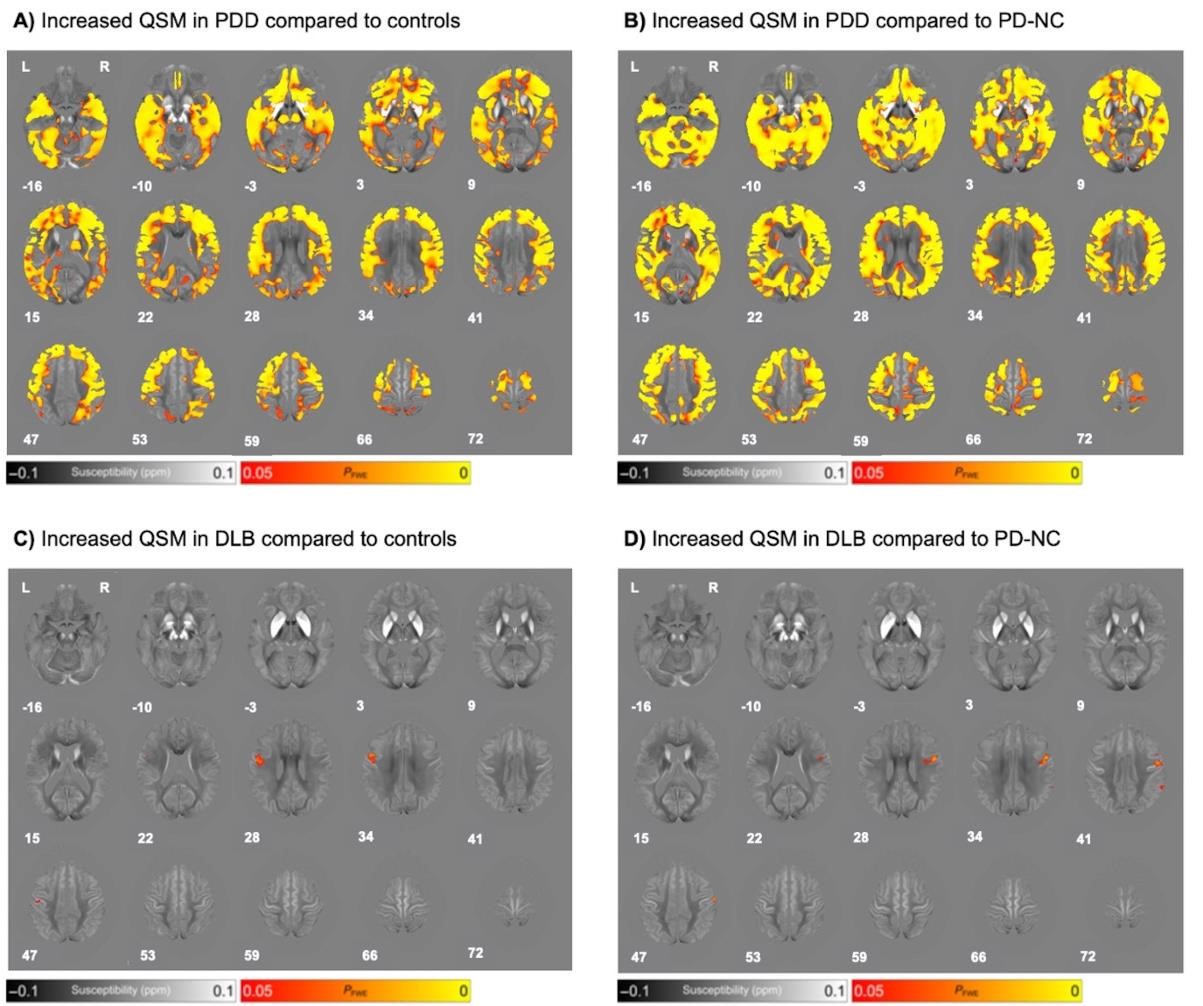


**Supplementary Figure 2. Group comparison of absolute QSM values in whole brain analysis**

S2A. PDD compared with controls. S2B. PDD compared with PD-NC.

S2C. DLB compared to controls

S2D. DLB compared to PD-NC

Red/yellow clusters represent voxels where a significant relationship was seen at FWEcorrected *P* < 0.05 (corrected for age and sex), with increased absolute magnetic susceptibility for PDD compared to controls (A); PDD compared to PD-NC (B), DLB compared to controls (C), and DLB compared to PD-NC (D).

Results are overlaid on the study-wise QSM template in MNI152 space, and numbers represent axial slice location in MNI152 space. Left side is shown on the left.

DLB, Dementia with Lewy bodies; PDD, Parkinson’s disease dementia; PD-NC, Parkinson’s disease with normal cognition; QSM, Quantitative susceptibility.


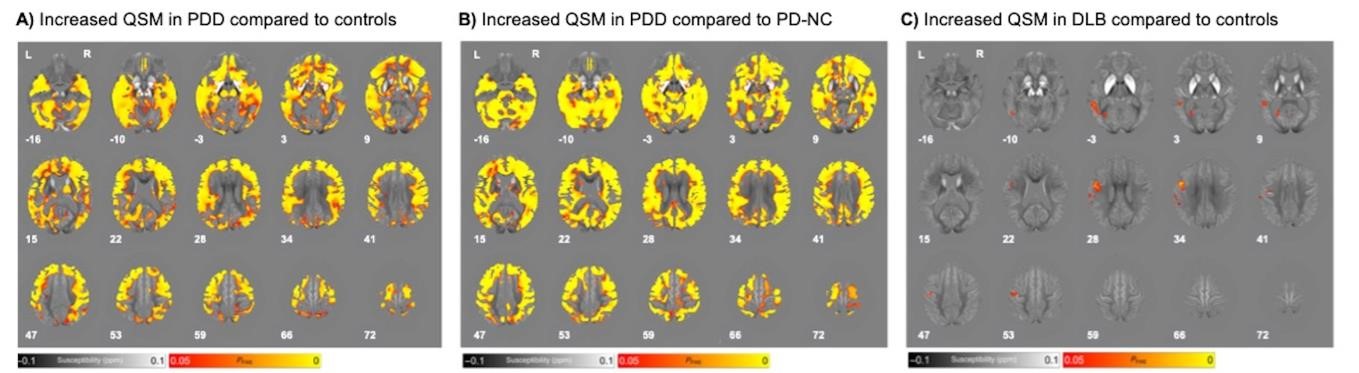


**Supplementary Figure 3. Group comparison of absolute QSM values in whole brain analysis, corrected**  **for TBV**

S2A. Increased QSM in PDD compared to controls

S2B. Increased QSM in PDD compared to PD-NC

S2C. Increased QSM in DLB compared to controls

No significant differences between DLB and PD-NC

S2A) Implicated regions include widespread bilateral frontal, temporal, parietal and occipital lobes. Additionally, the bilateral thalami, left hippocampus and left caudate.

S2B) Implicated regions include widespread bilateral frontal, temporal, parietal and occipital lobes. Additionally, left thalamus and right hippocampus

S2C) Implicated regions include the left postcentral and left lingual regions.

Results are overlaid on the study-wise QSM template in MNI152 space, and numbers represent axial slice location in MNI152 space. Left side is shown on the left. Red/yellow clusters represent voxels where a significant relationship was seen at FWE-corrected *P* < 0.05 (corrected for age, sex and TBV).

DLB, Dementia with Lewy bodies; PDD, Parkinson’s disease dementia; PD-NC, Parkinson’s disease with normal cognition; QSM, Quantitative susceptibility.

**ROI comparison between hemispheres**

Across all study participants (n=189), the superior parietal region had significantly different signed susceptibility between hemispheres, with the left side (Mean=0.0027; SD=0.0052) having greater susceptibility than the right side (Mean=0.00090; SD=0.0049) (t=3.38; P_FDR_=0.009) (**Supplementary Table 1a**). There were no group differences in susceptibility in either the left or right superior parietal region (**Supplementary table 1b**). We also tested the association between each superior parietal region and clinical measures separately (**Supplementary Table 1c**).

| **Supplementary Table 1a. Individual hemisphere QSM values for study population** |
| --- |
| **ROI Left Right t P P_FDR_**  NBM 0.14 (0.035) 0.14 (0.041) -0.79 0.43 0.65  Globus Pallidus 0.10 (0.023) 0.10 (0.025) 1.65 0.10 0.24  Caudate 0.044 (0.015) 0.043 (0.014) 0.67 0.50 0.67  Putamen 0.064 (0.022) 0.064 (0.023) 0.28 0.78 0.78  SNPr 0.048 (0.035) 0.049 (0.029) -0.46 0.65 0.75  SNPc 0.13 (0.040) 0.13 (0.039) -0.41 0.69 0.75  Thalamus -0.0050 (0.0050) -0.0037 (0.0055) -2.37 0.018 0.11  Hippocampus -0.0094 (0.0081) -0.0079 (0.0077) -1.86 0.064 0.24  Insula -0.0050 (0.0051) -0.0055 (0.0054) 0.96 0.34 0.65  Medial -0.0053 (0.0072) -0.0047 (0.0078) -0.36 0.39 0.65  Orbitofrontal  **Superior 0.0027 (0.0052) 0.00090 (0.0049) 3.38 0.00079 0.009**  **Parietal**  Lateral Occipital -0.0030 (0.0083) -0.0015 (0.0088) -1.65 0.099 0.24    QSM values are mean unsmoothed signed values, mean (SD) shown for whole study population    Unpaired t-tests were performed to compare hemisphere differences.    In **bold** result signifies FDR-corrected statistically significant result.    NBM, Nucleus Basalis of Meynert; SNpr, Substantia Nigra Pars Reticulata; SNpc, Substantia Nigra Pars Compacta |

| **Supplementary Table 1b. Regional mean signed magnetic susceptibilities**    **ROI DLB PDD PD-NC Controls Statistical (n=45) (n=21) (n=86) (n=37) comparison**    Left Superior 0.0042 0.0033 0.0023 0.0014 F=2.31;  Parietal (0.0048) (0.0093) (0.0041) (0.0047) P=0.078  Right Superior 0.013 0.00059 0.0015 -0.00087 F=2.24;  Parietal (0.0050) (0.0067) (0.0040) (0.0050) P=0.086  Bilateral Superior 0.0028 0.0022 0.0019 0.00020 F=2.55;  Parietal (0.0041) (0.0075) (0.0029) (0.0043) P=0.058    Mean (SD) of regional signed QSM values by group.  Group level comparison with ANOVAs, FDR corrected for multiple comparisons.    DLB, Dementia with Lewy bodies; PDD, Parkinson’s Disease Dementia; PD-NC, Parkinson’s disease with normal cognition |
| --- |

| **Supplementary Table 1c. Superior parietal QSM regression analysis against clinical measures**  **ROI MoCA Composite MDS-UPDRS MDS-UPDRS**  **cognitive Score total score motor score**  **LBD (n=66)**  Left Superior β=60.18 β=46.47 β=1.60 β=-57.83  Parietal (SE=97.38); (SE=48.78); (SE=566.91); (SE=316.24);  P=0.54 P=0.35 P=0.99 P=0.86  Right Superior β=51.03 β=17.72 β=566.00 β=343.00  Parietal (SE=113.93); (SE=46.11); (SE=658.35); (SE=366.95);  P=0.66 P=0.70 P=0.39 P=0.35  Bilateral Superior β=63.20 β=43.97 β=391.08 β=162.38  Parietal (SE=118.07); (SE=56.82); (SE=684.99); (SE=382.66);  P=0.59 P=0.44 P=0.57 P=0.67 **DLB (n=45)**  Left Superior **β=415.89 β=114.87 β=-2414.00** β=-873.85  Parietal **(SE=164.40); (SE=55.12); (SE=922.40);** (SE=558.01);  **P=0.015 P=0.04 P=0.01** P=0.13  Right Superior β=231.64 β=44.14 β=70.17 β=252.12  Parietal (SE=165.55); (SE=55.14); (SE=955.23); (SE=549.29);  P=0.17 P=0.43 P=0.94 P=0.65  Bilateral Superior **β=447.95 β=110.84** β=-1636.18 β=-432.59  Parietal **(SE=194.39); (SE=65.50);** (SE=1136.29); (SE=667.99);  **P=0.026 P=0.10** P=0.16 P=0.52  **PDD (n=21)**  Left Superior β=-143.20 **β=-213.66 β=1516.00** β=467.75  Parietal (SE=96.29); **(SE=104.83); (SE=413.80);** (SE=273.81);  P=0.16 **P=0.06 P=0.0019** P=0.11  Right Superior β=-164.13 β=-99.99 β=1359.11 β=559.60  Parietal (SE=141.32); (SE=104.97); (SE=722.37); (SE=402.86);  P=0.26 P=0.36 P=0.07 P=0.18  Bilateral Superior β=-190.81 β=-220.54 **β=1907.35** β=635.37  Parietal (SE=121.15); (SE=123.27); **(SE=527.17);** (SE=342.41);  P=0.13 P=0.10 **P=0.0021** P=0.081 |
| --- |
| Linear regressions were performed with regional mean signed QSM values as the independent variable and the clinical measure as the dependent variable, adjusting for age and sex.    DLB, Dementia with Lewy bodies; HVOT, Hooper Visual Organisation Test; MoCA, Montreal  Cognitive Assessment; MDS-UPDRS, Movement Disorders Society Unified Parkinson’s Disease  Rating Scale; NBM, Nucleus Basalis of Meynert; PDD, Parkinson’s disease dementia; SNpr,  Substantia Nigra Pars Reticulata; SNpc, Substantia Nigra Pars Compacta |

| **Supplementary Table 2. ROI signed QSM regression analysis against clinical measures in LBD** | | | | | | |  |  |  |  |
| --- | --- | --- | --- | --- | --- | --- | --- | --- | --- | --- |
| **ROI** | **MoCA** | **PFDR** | **Hooper** | **PFDR** | **Composite cognitive Score** | **PFDR** | **MDS-UPDRS**  **Total score** | **PFDR** | **MDS- UPDRS**  **Motor score** | **PFDR** |
| NBM | β=13.08  (SE=17.36);  P=0.45 | 0.71 | β=25.56  (SE=20.20);  P=0.21 | 0.72 | β=3.12  (SE=6.08);  P=0.61 | 0.80 | β=2.29  (SE=101.23);  P=0.98 | 0.98 | β=-24.50  (SE=56.40);  P=0.67 | 0.67 |
| Globus Pallidus | β=28.54  (SE=25.56);  P=0.27 | 0.71 | β=25.07  (SE=30.11);  P=0.41 | 0.72 | β=3.95  (SE=9,17);  P=0.67 | 0.80 | β=53.61  (SE=149.65);  P=0.72 | 0.79 | β=-41.24  (SE=83.43);  P=0.62 | 0.67 |
| Caudate | β=38.99  (SE=39.09);  P=0.32 | 0.71 | β=81.28  (SE=45.05);  P=0.08 | 0.48 | β=17.23  (SE=14.02);  P=0.22 | 0.75 | β=-316.42  (SE=225.12);  P=0.16 | 0.64 | β =-150.68  (SE=126.15);  P=0.24 | 0.58 |
| Putamen | β=22.66  (SE=25.15);  P=0.37 | 0.71 | β=32.78  (SE=29.40);  P=0.27 | 0.72 | β=7.13  (SE=8.73);  P=0.42 | 0.75 | β=-56.05  (SE=146.74);  P=0.70 | 0.79 | β =-37.68  (SE=81.83);  P=0.65 | 0.67 |
| SNPr | β =-12.22  (SE=18.43);  P=0.51 | 0.71 | β=-1.50  (SE=21.70);  P=0.95 | 0.95 | β=-6.75  (SE=6.86);  P=0.33 | 0.75 | **β=336.72 (SE=98.46);**  **P=0.0011** | **0.01** | β=159.45 (SE=56.37);  P=0.00629 | 0.08 |
| SNPc | β =-26.38  (SE=16.05);  P=0.11 | 0.66 | Β=-5.74  (SE=19.22);  P=0.77 | 0.92 | β=-8.30  (SE=5.56);  P=0.14 | 0.75 | β=205.46  (SE=91.50);  P=0.028 | 0.17 | β=85.09  (SE=51.98);  P=0.11 | 0.36 |
| Thalamus | β=51.12  (SE=111.09);  P=0.65 | 0.71 | β=105.61  (SE=129.84);  P=0.42 | 0.72 | β=0.86  (SE=38.39);  P=0.98 | 0.98 | β=-551.16  (SE=642.01);  P=0.39 | 0.76 | β=-556.20  (SE=353.36);  P=0.58 | 0.36 |
| Hippocampus | β=89.77  (SE=82.07;  P=0.28 | 0.71 | β=40.79  (SE=97.05);  P=0.68 | 0.91 | β=32.47  (SE=33.63;  P=0.34 | 0.75 | β=386.10  (SE=478.35);  P=0.42 | 0.76 | β=147.42  (SE=267.65);  P=0.52 | 0.67 |
| Insula | β=-251.28  (SE=124.83);  P=0.049 | 0.58 | β=-381.00  (SE=143.17);  P=0.0099 | 0.12 | β=-73.43  (SE=46.71);  P=0.12 | 0.75 | β=479.56  (SE=745.25);  P=0.52 | 0.76 | β=270.17  (SE=415.80);  P=0.49 | 0.67 |
| Medial  Orbitofrontal | β=-9.08  (SE=78.23);  P=0.91 | 0.91 | β=-12.64;  (SE=91.76);  P=0.89 | 0.95 | β=-13.43  (SE=30.02);  P=0.66 | 0.80 | β=-366.57  (SE=451.68);  P=0.42 | 0.76 | β=-177.04  (SE=252.37);  P=0.49 | 0.67 |
| Superior Parietal β=63.20 0.71 β=-132.23 0.72 β=43.97 0.75 β=391.08 0.76 β=162.38 0.67  (SE=118.07); (SE=137.79); (SE=56.82); (SE=684.99); (SE=382.66);  P=0.59 P=0.34 P=0.44 P=0.57 P=0.67  Lateral Occipital β=59.22 0.71 β=-61.64 0.87 β=4.11 0.98 β=423.95 0.76 β=511.63 0.36  (SE=95.41); (SE=111.98); (SE=32.84); (SE=552.80); (SE=303.02);  P=0.54 P=0.58 P=0.90 P=0.45 P=0.10 | | | | | | | | | | |
| Linear regressions were performed with regional mean signed QSM values as the independent variable and the clinical measure as the dependent variable, adjusting for age and sex.    LBD, Lewy body Dementia; HVOT, Hooper Visual Organisation Test MoCA, Montreal Cognitive Assessment; MDS-UPDRS, Movement Disorders Society Unified Parkinson’s Disease Rating Scale.    In **bold** result signifies FDR-corrected statistically significant result. | | | | | | | | | | |

| **Supplementary Table 3. ROI signed QSM regression analysis against clinical measures in DLB** | | | | | | |  |  |  |  |
| --- | --- | --- | --- | --- | --- | --- | --- | --- | --- | --- |
| **ROI** | **MoCA** | **PFDR** | **Hooper** | **PFDR** | **Composite cognitive Score** | **PFDR** | **UPDRS Total** | **PFDR** | **UPDRS Motor** | **PFDR** |
| NBM | β=3.98  (SE=25.10);  P=0.87 | 0.98 | β=11.19  (SE=27.52);  P=0.69 | 0.75 | β=-0.39 (SE=8.23);  P=0.96 | 0.96 | β=-13.80  (SE=141.50);  P=0.92 | 0.92 | β=-41.94  (SE=81.32);  P=0.61 | 0.79 |
| Globus Pallidus | β=45.02  (SE=38.54);  P=0.25 | 0.56 | β=22.18  (SE=42.89);  P=0.61 | 0.75 | β=8.14  (SE=12.78);  P=0.53 | 0.91 | β=-206.14  (SE=218.49);  P=0.35 | 0.70 | β=-173.62  (SE=124.39);  P=0.17 | 0.41 |
| Caudate | β=10.32  (SE=59.82);  P=0.86 | 0.98 | β=25.60  (SE=65.60);  P=0.70 | 0.75 | β=3.08  (SE=19.62);  P=0.88 | 0.96 | β=-532.09  (SE=326.96);  P=0.11 | 0.44 | β=-290.33  (SE=189.11);  P=0.13 | 0.41 |
| Putamen | β=-1.30  (SE=42.42);  P=0.98 | 0.98 | β=-15.18  (SE=46.53);  P=0.75 | 0.75 | β=-6.33  (SE=13.88);  P=0.65 | 0.92 | β=-46.71  (SE=239.04);  P=0.85 | 0.92 | β=-36.63  (SE=137.74);  P=0.79 | 0.79 |
| SNPr | β=-51.70  (SE=29.30);  P=0.085 | 0.34 | β=-23.14  (SE=33.19);  P=0.49 | 0.75 | β=-15.94  (SE=9.65);  P=0.11 | 0.44 | β=366.85  (SE=161.49);  P=0.028 | 0.34 | β=188.14;  (SE=94.31);  P=0.053 | 0.41 |
| SNPc | β=-44.03  (SE=23.89);  P=0.07 | 0.34 | β=-24.62  (SE=27.03);  P=0.37 | 0.75 | β=-15.89 (SE=7.77);  P=0.0472 | 0.44 | β=258.04  (SE=134.22);  P=0.0615 | 0.37 | β=112.72  (SE=78.84);  P=0.16 | 0.41 |
| Thalamus | β=2.93  (SE=131.20);  P=0.98 | 0.98 | β=63.31  (SE=143.77);  P=0.66 | 0.75 | β=-12.68  (SE=42.98);  P=0.77 | 0.92 | β=-468.97  (SE=736.02);  P=0.53 | 0.91 | β=-518.45  (SE=418.63);  P=0.22 | 0.44 |
| Hippocampus | β=180.91  (SE=129.12);  P=0.17 | 0.51 | β=108.12  (SE=144.19);  P=0.46 | 0.75 | β=53.65  (SE=42.53);  P=0.21 | 0.63 | β=257.37  (SE=744.06);  P=0.73 | 0.92 | β=177.58  (SE=428.66);  P=0.68 | 0.44 |
| Insula | β=-228.40  (SE=209.30);  P=0.28 | 0.56 | β=-177.07  (SE=231.53);  P=0.45 | 0.75 | β=-43.66  (SE=69.29);  P=0.53 | 0.91 | β=254.84  (SE=1196.18);  P=0.83 | 0.92 | β=189.44  (SE=689.30);  P=0.79 | 0.79 |
| Medial  Orbitofrontal | β=-92.43  (SE=131.36);  P=0.49 | 0.84 | β=-141.89  (SE=143.44);  P=0.33 | 0.75 | β=-31.54  (SE=43.06);  P=0.47 | 0.91 | β=-299.97  (SE=743.51);  P=0.69 | 0.92 | β=-118.24  (SE=429.06);  P=0.78 | 0.79 |
| Superior Parietal β=447.95 0.32 β=96.25 0.75 β=110.84 0.44 β=-1636.18 0.48 β=-432.59; 0.79  (SE=194.39); (SE=226.41); (SE=65.50); (SE=1136.29); (SE=667.99);  P=0.0263 P=0.67 P=0.0982 P=0.16 P=0.52  Lateral Occipital β=32.85 0.98 β=-63.47 0.75 β=13.63 0.92 β=764.68 0.70 β=727.87 0.41  (SE=127.87); (SE=140.21); (SE=41.91); (SE=711.47); (SE=400.04);  P=0.80 P=0.65 P=0.75 P=0.29 P=0.076 | | | | | | | | | | |
| Linear regressions were performed with regional mean signed QSM values as the independent variable and the clinical measure as the dependent variable, adjusting for age and sex.    DLB, Dementia with Lewy bodies; HVOT, Hooper Visual Organisation Test; MoCA, Montreal Cognitive Assessment; MDS-UPDRS, Movement Disorders  Society Unified Parkinson’s Disease Rating Scale; NBM, Nucleus Basalis of Meynert; SNpr, Substantia Nigra Pars Reticulata; SNpc, Substantia Nigra Pars Compacta | | | | | | | | | | |

| **Supplementary Table 4. ROI signed QSM regression analysis against clinical measures in PDD** | | | | | | |  |  |  |  |
| --- | --- | --- | --- | --- | --- | --- | --- | --- | --- | --- |
| **ROI** | **MoCA** | **PFDR** | **Hooper** | **PFDR** | **Composite cognitive Score** | **PFDR** | **UPDRS Total** | **PFDR** | **UPDRS Motor** | **PFDR** |
| **NBM** | β=22.24  (SE=21.66);  P=0.32 | 0.55 | β=39.01  (SE=29.94);  P=0.21 | 0.50 | β=7.65  (SE=8.12);  P=0.36 | 0.74 | β=14.27  (Se=120.67);  P=0.91 | 0.91 | β=10.46  (SE=64.58);  P=0.87 | 0.87 |
| **Globus Pallidus** | β=5.31  (SE=32.72);  P=0.87 | 0.95 | β=39.83  (SE=45.03): P=0.39 | 0.65 | β=3.52  (SE=13.33);  P=0.80 | 0.90 | β=190.94  (SE=170.93);  P=0.28 | 0.50 | Β=43.99  (SE=94.21);  P=0.65 | 0.82 |
| **Caudate** | β=58.05  (SE=47.12);  P=0.24 | 0.50 | β=136.26  (SE=50.77);  P=0.039 | 0.23 | β=35.39  (SE=17.34);  P=0.061 | 0.44 | β=-292.19  (SE=256.37);  P=0.27 | 0.50 | β=-96.52  (SE=140.47);  P=0.50 | 0.75 |
| **Putamen** | β=33.37  (SE=27.98);  P=0.25 | 0.50 | β=56.73  (SE=38.59): P=0.16 | 0.48 | β=13.35  (SE=9.91);  P=0.20 | 0.60 | β=-129.97  (SE=154.34);  P=0.41 | 0.61 | β=-83.58  (SE=81.87);  P=0.32 | 0.62 |
| **SNPr** | β=15.31  (SE=27.94);  P=0.59 | 0.80 | β=16.46  (SE=39.44);  P=0.68 | 0.89 | β=2.29  (SE=11.83);  P=0.85 | 0.90 | β=231.64  (SE=141.70);  P=0.12 | 0.48 | β=100.52  (SE=77.88);  P=0.21 | 0.62 |
| **SNPc** | β=-11.33  (SE=21.10);  P=0.60 | 0.80 | β=23.88  (SE=29.36);  P=0.43 | 0.65 | β=3.69  (SE=8.04);  P=0.65 | 0.90 | β=43.71  (SE=114.58);  P=0.71 | 0.77 | β=14.82  (SE=61.50);  P=0.81 | 0.87 |
| **Thalamus** | β=354.63  (SE=252.42);  P=0.18 | 0.50 | β=318.38  (SE=369.78);  P=0.40 | 0.65 | β=95.13  (SE=101.98);  P=0.37 | 0.74 | β=-1698.65  (SE=1392.79);  P=0.24 | 0.50 | β=-1190.46  (SE=722.02);  P=0.12 | 0.48 |
| **Hippocampus** | β=-2.08  (SE=94.45);  P=0.98 | 0.98 | β=-45.12  (SE=132.41);  P=0.74 | 0.89 | β=-6.50  (SE=53.16);  P=0.90 | 0.90 | β=542.80  (SE=493.58);  P=0.29 | 0.50 | β=116.05  (SE=272.04);  P=0.68 | 0.82 |
| **Insula** | β=-245.00  (SE=138.16);  P=0.94 | 0.50 | β=-550.18 (SE=164.14);  P=0.00378 | 0.45 | β=-94.44  (SE=54.87);  P=0.11 | 0.44 | β=1402.47  (SE=738.83);  P=0.075 | 0.45 | β=700.83  (SE=400.92);  P=0.0985 | 0.48 |
| **Medial**  **Orbitofrontal** | β=34.14  (SE=89.53);  P=0.71 | 0.85 | β=27.40  (SE=126.29);  P=0.83 | 0.91 | β=-19.66  (SE=41.27);  P=0.64 | 0.90 | β=--325.47  (SE=479.81);  P=0.51 | 0.61 | β=-240.23  (SE=253.73);  P=0.36 | 0.62 |
| **Superior Parietal** β=-190.81 0.50 β=-279.21 0.48 β=-220.54 0.44 **β=1907.35 0.03** β=635.37 0.48 (SE=121.15); (SE=169.37); (SE=123.27); **(SE=527.17);** (SE=342.41);  P=0.13 P=0.12 P=0.095 **P=0.0021** P=0.081  **Lateral Occipital** β=210.63 0.50 β=-13.18 0.95 β=9.92 0.90 β=556.36 0.61 β=479.33 0.62  (SE=141.39); (SE=211.43); (SE=56.98); (SE=801.77); (SE=419.47);  P=0.16 P=0.95 P=0.86 P=0.50 P=0.27 | | | | | | | | | | |
| Linear regressions were performed with regional mean signed QSM values as the independent variable and the clinical measure as the dependent variable, adjusting for age and sex    PDD, Parkinson’s disease Dementia; HVOT, Hooper Visual Organisation Test; MoCA, Montreal Cognitive Assessment; MDS-UPDRS, Movement Disorders  Society Unified Parkinson’s Disease Rating Scale; NBM, Nucleus Basalis of Meynert; SNpr, Substantia Nigra Pars Reticulata; SNpc, Substantia Nigra Pars Compacta    In **bold** result signifies FDR-corrected statistically significant result | | | | | | | | | | |

| **Supplementary Table 5. Whole SN mean QSM values reported in the current study and by Chen et al^5^** |
| --- |
| DLB (our PDD (our iRBD (Chen, MCI-LB pDLB (Chen, study) study) 2021) (Chen, 2021) 2021)  N = 45 N = 21 N = 11 N = 15 N = 36    Substantia nigra 0.092 (0.03) 0.11 (0.03) 0.10 (0.02) 0.096 (0.03) 0.11 (0.03) |
| Means (SDs) are reported.  SN, substantia nigra; DLB dementia with Lewy bodies; PDD, Parkinson’s disease dementia; iRBD, idiopathic  REM sleep behaviour disorder; MCI-LB, mild cognitive impairment with Lewy bodies; pDLB, probable DLB. |

| **Supplementary Table 6. Cross-study group comparison of whole SN mean QSM values between the current study and Chen et al.^5^** |
| --- |
| iRBD (Chen, 2021) MCI-LB (Chen, 2021) pDLB (Chen, 2021)    DLB (our study) *ns ns pDLB > DLB*  T = 0.78 T = 0.40 T = 2.16 p = 0.44 p = 0.69 **p = 0.034**  pFDR = 0.66 pFDR = 0.79 pFDR = 0.21    PDD (our study) *ns ns ns*  T = 1.11 T = 1.50 T = 0.27 p = 0.28 p = 0.14 p = 0.79  pFDR = 0.55 pFDR = 0.43 pFDR = 0.79 |
| SN, substantia nigra; DLB dementia with Lewy bodies; PDD, Parkinson’s disease dementia; iRBD, idiopathic  REM sleep behaviour disorder; MCI-LB, mild cognitive impairment with Lewy bodies; pDLB, probable DLB. |

| **Supplementary Table 7. Location and peak significance of VBM group analyses.** |
| --- |
| **Comparison Region MNI T scores P_FWE_**  **Coordinates x y z**  **LBD < Controls**  Left parahippocampal gyrus -18 -1 -16 5.55 0.005  Right inferior temporal gyrus 49 1 -45 4.74 0.009  Right precentral gyrus 57 -3 37 4.31 0.013  Left middle frontal gyrus -33 17 44 6.09 0.005  Right precuneus cortex 11 -61 16 4.19 0.022    **DLB < Controls**  Right inferior temporal gyrus 56 -8 -34 5.27 0.011  Left inferior temporal gyrus -49 -7 -36 5.12 0.0018  Left parahippocampal gyrus -16 -1 -15 5.59 0.0014  Right parahippocampal gyrus 21 -1 -14 4.75 0.040  Left temporal pole -33 17 44 4.57 0.039    **PDD < Controls**  Right precentral gyrus 55 -3 40 4.78 0.015  Left middle frontal gyrus -32 17 50 5.29 0.023  Right intracalcarine cortex 16 -71 15 3.98 0.048    **LBD < PD-NC**  Right insular cortex 42 -14 4 3.77 <0.0001  Left parahippocampal gyrus -34 23 46 3.92 <0.0001  Paracingulate gyrus 0 -2 -15 3.93 <0.0001  Right parahippocampal gyrus 26 1 -17 4.24 <0.0001  Left inferior temporal gyrus -50 -1 -25 4.21 <0.0001  Left temporal pole -21 13 -37 4.29 <0.0001    **DLB < PD-NC**  Left parahippocampal gyrus -19 -7 -24 4.71 0.001    **PDD < PD-NC**  Right precentral gyrus 50 -6 37 4.69 0.002  Left parietal operculum cortex -53 -38 24 3.44 0.037  Left inferior cerebellar grey matter -45 -65 -57 3.41 0.037 Left inferior cerebellar grey matter -18 -50 -58 2.86 0.042 |
| This table shows the comparison, region, MNI co-ordinates, *t* scores and the corrected voxel-level significance (*p*_FWE_).  < indicates greater atrophy, eg LBD < controls signifies greater atrophy in the LBD group.  Signficant clusters reported at  *p*_FWE_ < 0.05, extent threshold (voxels) = 100.  DLB, Dementia with Lewy bodies; LBD, Lewy body Dementia; PD-NC, Parkinson’s disease with normal cognition; PDD, Parkinson’s Disease Dementia |

| **Supplementary Table 8. Location and peak significance of VBM clinical correlations** | | | | |  |  |
| --- | --- | --- | --- | --- | --- | --- |
| **Association** | **Region** | **MNI Coordinates x y z** | | | **T scores** | **PFWE** |
| **LBD**    ↑ Atrophy ↓ Composite cognitive score | Left inferior cerebellar grey matter | -18 | -68 | -60 | 4.43 | 0.008 |
|  | Right inferior cerebellar grey matter | 38 | -58 | -60 | 3.93 | 0.018 |
|  | Right superior temporal gyrus | 47 | -23 | 0 | 3.92 | 0.035 |
|  | Right superior cerebellar grey matter | 28 | -49 | -26 | 3.42 | 0.040 |
|  | Left precentral gyrus | -55 | -3 | 13 | 3.42 | 0.044 |
|  | Right lateral occipital cortex | 49 | -83 | 6 | 3.01 | 0.047 |
|  | Right middle temporal gyrus | 61 | -46 | -6 | 3.44 | 0.044 |
|  | Right angular gyrus | 65 | -51 | 29 | 3.58 | 0.045 |
|  | Right supramarginal gyrus | 69 | -27 | 25 | 3.58 | 0.046 |
|  | Right supramarginal gyrus | 56 | -42 | 13 | 2.97 | 0.049 |
| ↑ Atrophy ↓ MoCA | Left inferior cerebellar grey matter | -20 | -66 | -60 | 4.40 | 0.005 |
|  | Right cingulate gyrus | 2 | -31 | 49 | 4.46 | 0.027 |
|  | Right inferior cerebellar grey matter | 32 | -66 | -56 | 3.43 | 0.029 |
|  | Right superior frontal gyrus | 1 | 33 | 44 | 3.42 | 0.046 |
|  | Left paracingulate gyrus | -2 | 41 | 30 | 2.99 | 0.049 |
| ↑ Atrophy ↑ UPDRS | Left inferior cerebellar grey matter | -44 | -52 | -58 | 5.17 | 0.019 |
|  | Right inferior cerebellar grey matter | 4 | -87 | -33 | 3.82 | 0.042 |
|  | Left occipital fusiform gyrus | -21 | -72 | -21 | 4.51 | 0.041 |
| **DLB**    ↑ Atrophy ↓ MoCA | Right inferior cerebellar grey matter | -8 | -81 | -46 | 4.39 | 0.034 |
| **PDD**    ↑ Atrophy ↓ Composite cognitive score | Right frontal orbital cortex | 17 | 5 | -20 | 7.53 | 0.026 |
|  | Left insular cortex | -34 | 8 | -18 | 4.12 | 0.046 |
|  | Left temporal fusiform cortex | -25 | -4 | -50 | 7.64 | 0.021 |
|  | Right temporal fusiform cortex | 26 | -2 | -50 | 8.15 | 0.021 |
|  | Right temporal fusiform cortex | 33 | -21 | -35 | 4.99 | 0.043 |
|  | Left subcallosal cortex | -10 | 19 | -9 | 3.94 | 0.049 |
|  | Right caudate | 20 | -21 | 13 | 4.91 | 0.047 |
|  | Right temporal pole | 30 | 15 | -47 | 4.36 | 0.048 |
| ↑ Atrophy ↓ MoCA | Left temporal fusiform cortex | -30 | -4 | -44 | 6.22 | 0.01 |
|  | Right superior parietal lobule | 40 | -36 | 47 | 4.58 | 0.036 |
|  | Left precentral gyrus | -8 | -22 | 64 | 5.25 | 0.035 |
|  | Left supramarginal gyrus | -44 | -36 | 39 | 4.04 | 0.044 |
|  | Left angular gyrus | -48 | -49 | 25 | 4.31 | 0.044 |
|  | Right paracingulate gyrus | 4 | 45 | 0 | 3.77 | 0.049 |
|  | Left precentral gyrus | -54 | 0 | 13 | 4.71 | 0.047 |
|  | Left middle temporal gyrus | -52 | -53 | 6 | 3.50 | 0.049 |
| Left superior parietal lobule -11 -55 73 4.23 0.047  Left central opercular cortex -56 -23 20 4.44 0.048 | | | | | | |
| This table shows the comparison, region, MNI co-ordinates, *t* scores and the corrected voxel-level significance (*p*_FWE_). ↑ atrophy indicates more atrophy, i.e. ↑ Atrophy ↓ MoCA indicates increasing atrophy with decreasing MoCA  Signficant clusters reported at  *p*_FWE_ < 0.05, extent threshold (voxels) = 100.  DLB, Dementia with Lewy bodies; LBD, Lewy body Dementia; PD-NC, Parkinson’s disease with normal cognition; PDD, Parkinson’s Disease Dementia; MoCA, Montreal cognitive assessment; UPDRS, Unified Parkinson’s disease rating scale | | | | | | |

**Supplementary references**

1. Thomas GEC, Leyland LA, Schrag AE, Lees AJ, Acosta-Cabronero J, Weil RS. Brain iron deposition is linked with cognitive severity in Parkinson's disease. J Neurol Neurosurg Psychiatry. 2020;91(4):418-25.

1. Chen Q, Boeve BF, Forghanian-Arani A, Senjem ML, Jack CR, Jr., Przybelski SA, et al. MRI quantitative susceptibility mapping of the substantia nigra as an early biomarker for Lewy body disease. J Neuroimaging. 2021;31(5):1020-7.

1. Li X, Chen L, Kutten K, Ceritoglu C, Li Y, Kang N, et al. Multi-atlas tool for automated segmentation of brain gray matter nuclei and quantification of their magnetic susceptibility. Neuroimage. 2019;191:337-49.

1. Klein A, Tourville J. 101 labeled brain images and a consistent human cortical labeling protocol. Front Neurosci. 2012;6:171.

1. Chen Q, Boeve BF, Forghanian-Arani A, Senjem ML, Jack CR, Przybelski SA, et al. MRI quantitative susceptibility mapping of the substantia nigra as an early biomarker for Lewy body disease. Journal of Neuroimaging. 2021;31(5):1020-7.
